# Disseminated *Staphylococcus aureus* infection after scarification wet cupping therapy: a case report and literature review

**DOI:** 10.1186/s12906-023-03932-x

**Published:** 2023-03-30

**Authors:** You-yang Wang, Hong-wei Fan, Xiao-ming Huang, Yang Jiao

**Affiliations:** 1 Department of General Internal Medicine (General Practice), Peking Union Medical College Hospital, Chinese Academy of Medical Sciences & Peking Union Medical College, No. 1, Shuaifuyuan, Wangfujing St., Beijing, 100730 China; 2 Department of Infectious Diseases, Peking Union Medical College Hospital, Chinese Academy of Medical Sciences & Peking Union Medical College, Beijing, China

**Keywords:** Complementary medical therapy, Infection, *Staphylococcus aureus*, Wet cupping, Case report

## Abstract

**Background:**

Cupping therapy is a complementary and alternative medical therapy used especially in pain management. It is generally considered a safe procedure, but complications, including life-threatening infection, may still occur. Understanding these complications is essential to safe and evidence-based use of cupping in practice.

**Case presentation:**

Here we report a rare case of disseminated *Staphylococcus aureus* infection after cupping therapy. After wet cupping, a 33-year-old immunocompetent woman developed fever, myalgia, and a productive cough accompanied by acute liver and kidney injury, iliopsoas abscess, and gastrointestinal bleeding. The patient was treated successfully with cefmetazole plus levofloxacin after microbiological and antimicrobial sensitivity testing.

**Conclusions:**

Though rarely reported, clinicians, practitioners of cupping therapy, and patients should be aware of the risk of infection after cupping therapy. High hygiene standards are recommended for cupping therapy, even in immunocompetent individuals.

## Background

Cupping therapy, a traditional and complementary medicine (TCM) originating in the Middle East and East Asia, has become popular worldwide. Cupping can be classified into two main types: dry and wet cupping (*Hijama*). Dry cupping uses cups to create negative pressure on the skin through suction, while wet cupping is relatively invasive and involves scarification with needles or surgical blades before cupping, such that blood is drawn into the cup. Cupping therapy has particularly been used in pain management, especially for musculoskeletal pain [1–3] and headache [4, 5], as well as for respiratory diseases, acne, and facial paralysis [6–8]. There are several proposed mechanisms of action of cupping therapy including mimicking an artificial kidney, diffuse noxious inhibitory control, and release of nitric oxide [9].

Cupping therapy is generally considered a safe intervention with rare and mild adverse events. Scar formation, burns, headache, pruritis, dizziness, anemia, and panniculitis are relatively frequently observed after cupping therapy, but these are usually mild and self-limiting [7, 10, 11]. However, compromise of the dermal barrier, particularly with wet cupping, increases the risk of skin infection and abscess formation, and several infective adverse events have been reported with the procedure. Here we present a case of disseminated *Staphylococcus aureus* infection after wet cupping. Our case provides the opportunity to systematically review other cases of infection secondary to cupping and to discuss the risk of infection from cupping therapy.

## Case presentation

A 33-year-old woman was admitted to our hospital due to an 11-day history of fever, myalgia, cough, and sputum production. Eleven days prior to admission, she had received wet cupping to treat an accidental sprain of her upper right thigh. Apart from a reported penicillin allergy, her personal and medical history were unremarkable, and there was no previous history of dermatopathology. A traditional Chinese medical practitioner had applied wet cupping, including scarification with needles and suctioning with plastic cups to her right thigh, with only a small amount of blood loss reported. The practitioner disinfected the cupping sites with medical alcohol and used disposable sterile needles, but it was unclear whether any other medical devices and processes used during the procedure maintained strict sterility.

On the night of cupping, she developed an erythematous rash all over her body and intermittent fever with a peak temperature (T_max_) of 39.9℃. By five days after cupping, she had developed pain and stiffness of the right lower limb and proximal upper limbs bilaterally, with non-pitting edema in her feet and yellow, tea-like urine. Severe pain led to limitation of limb motion. On day 11 after cupping, she developed a productive cough.

Upon admission to hospital, she was febrile but her vital signs were otherwise unremarkable. Hematological examination showed an elevated white blood cell count (23.0 × 10^9^/L) with neutrophilia and mild anemia (hemoglobin 98 g/L), while blood chemistry analysis revealed elevated liver enzymes and creatinine with hypoalbuminemia and bilirubinemia. There was a pronounced inflammatory response (erythrocyte sedimentation rate 118 mm/h; high-sensitivity C-reactive protein, 42.98 mg/L). Urinalysis showed hematuria, leukocyturia, and mild proteinuria. Echocardiogram revealed a low left ventricular ejection fraction (57%), and no vegetations were detected. A CT scan revealed patchy high-density shadows in the lungs bilaterally with pleural effusions and a multilocular low-density shadow in the right iliopsoas, whose body surface projection was located near the wet cupping sites. Puncture of the iliac fossa was consistent with bacterial abscess. She was diagnosed with disseminated infection and treated empirically with antibiotics and steroids. However, her symptoms did not improve, and she developed hematochezia and hematemesis, resulting in severe anemia (minimum hemoglobin 35 g/L).

**Fig. 1:**
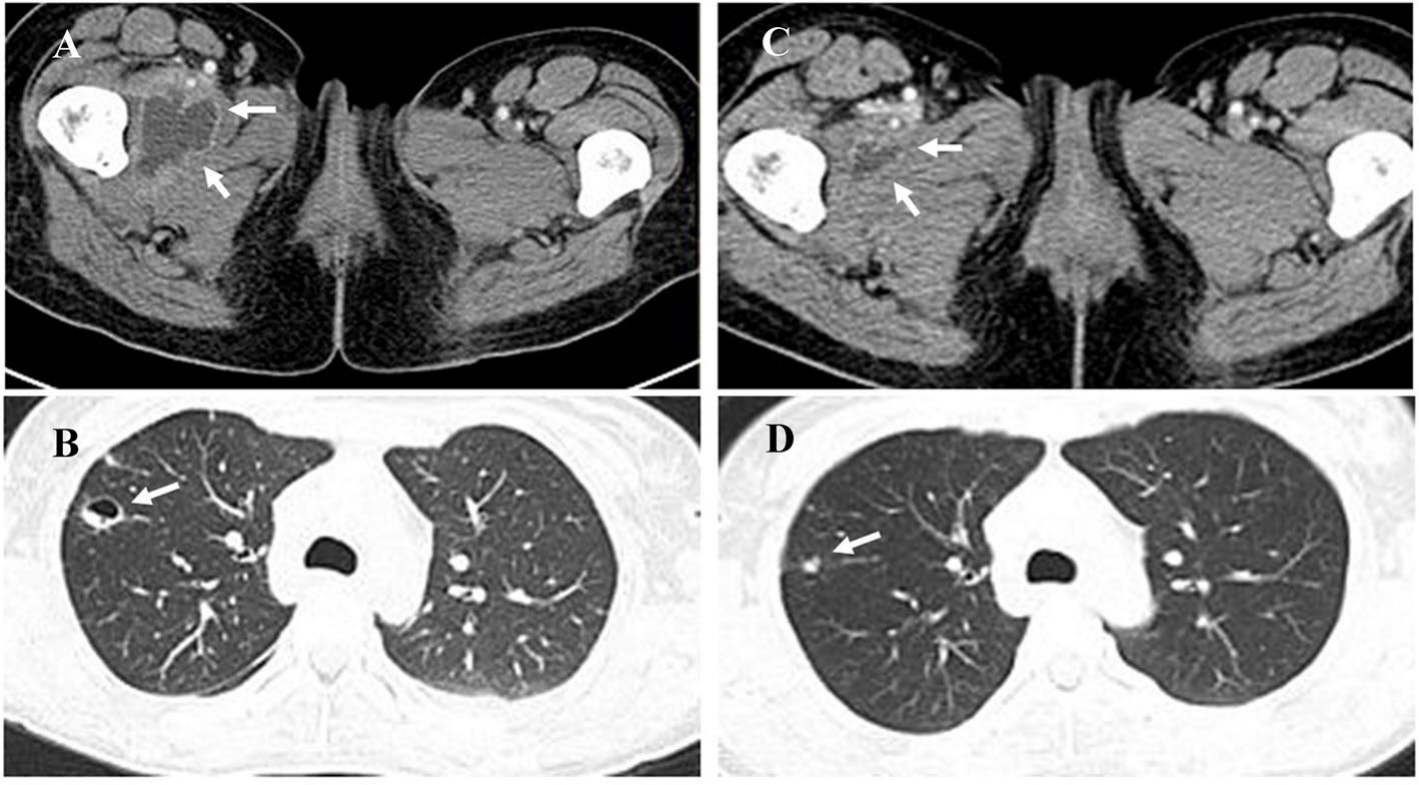
Appearance of CT scan acquired before and after cefmetazole plus levofloxacin treatment, i.e., on day 15 and day 30 after admission. The CT scan acquired before using cefmetazole plus levofloxacin treatment (1**A**, 1**B**) showed a multilocular low-density shadow in the right iliopsoas muscle (white arrows in 1**A**), and a high-density shadow with newly formed cavitation in the right lung (white arrow in 1**B**), suggesting multiple abscess formation. The CT scan acquired after 12 days of cefmetazole plus levofloxacin (1**C**, 1**D**) showed that both the right iliac muscle (white arrows in 1**C**) and right lung (white arrow in 1**D**) abscesses had decreased in size

Strains of methicillin-sensitive *Staphylococcus aureus* (MSSA) were isolated from her blood and the antibiotic was changed to vancomycin. Within seven days, the gastrointestinal bleeding stopped, and her hematology and biochemistry tests returned to normal except for moderate anemia after a blood transfusion. Despite the remission of other symptoms, the patient’s myalgia and fever persisted (T_max_ 38.2℃). Imaging showed expansion of the iliopsoas abscess with a large pelvic effusion and newly formed cavitation in the high-density shadows in the lungs (Fig. 1A, B).

The antibiotic was changed to cefmetazole 2 g every 12 h and levofloxacin 0.5 g four times a day based on the result of drug sensitivity testing. Twelve days after the start of cefmetazole plus levofloxacin, her temperature returned to normal and the myalgia significantly remitted. Multiple blood and sputum cultures were all negative. A CT scan showed a decrease in the size of the right iliac muscle and bilateral lesions, and the pelvic and pleural effusions had both resolved (Fig. 1C, D). She was discharged from hospital with a prescription to finish the overall 8-week course of antibiotics. At follow-up after treatment, the patient had completely recovered, and there was no residual myalgia nor abnormal imaging findings.

## Literature review

Cupping therapy is a popular TCM, especially in the Middle East and East Asia. It has reported benefits in pain management [3] and treating respiratory diseases [7] and dermatoses [8]. Previous clinical trials and meta-analysis have mainly focused on its efficacy [1, 3–5, 7, 9, 12, 13], but less attention has been paid to adverse events (AEs). Only a few reviews have focused on infection associated with cupping therapy [10, 11].

Here we present a case of disseminated *Staphylococcus aureus* infection resulting from wet cupping. Searching the PubMed and Chinese National Knowledge Infrastructure (CNKI) databases with the keywords “cupping therapy” and “infection” for case reports in the English and Chinese languages respectively, we found ten other cases reported as infection secondary to cupping therapy (Table 1) [14–23]. Cases were an even mix of males and females aged between 12 and 59 years. All patients were immunocompetent with no specific risk factors. Cupping therapy on the back induced epidural abscess formation in four cases, cupping on the limbs induced necroses, septic arthritis, or ulcers in four cases, cupping on the sternoclavicular joint induced osteomyelitis in one case, and cupping on the abdomen induced ulcer formation in one case.

**Table 1:** Cases reported in the literature

Study	Country	Sex	Age	Reason for cupping	Cupping method	Cupping points	Diagnosis	Organism	Symptoms	Treatment	Outcome
Alajmi, 2021 [14]	Saudi Arabia	M	35	N/A	Wet	Bilateral lower extremities	Necrotizing fasciitis	*Pseudomonas spp.*	Pain, swelling, skin lesions, fever	Vancomycin + meropenem, ICU + intubation + mechanical ventilation	Dead
Hasbani, 2020 [15]	Lebanon	M	46	N/A	Wet	Knee and periphery	Septic arthritis	MSSA	Pain, swelling	Aspiration and antibiotics	Complete resolution
Wang, 2019 [16]	China	F	12	Low back strain	Dry	Lower pack	Discitis and epidural abscess	*Stenotrophomonas maltophilia*	Pain, fever	Piperacillin/tazobactam → vancomycin → rifampicin + doxycycline + amikacin → sulfamethoxazole-trimethoprim + minocycline	Complete resolution
Xu, 2019 [17]	China	F	52	Sternoclavicular joint pain	Wet	Sternoclavicular joint and periphery	Osteomyelitis	N/A	Pain, swelling, fever	Surgical debridement + vancomycin	Complete resolution
Lu, 2017 [18]	China	M	34	N/A	Wet	Dorsum of foot	Skin necrosis	N/A	Pain, swelling, fever	Clindamycin → surgical debridement + piperacillin/tazobactam	Complete resolution
Yao, 2016 [19]	China	M	54	Chronic pain in neck and shoulders	Wet	Posterior nuchal and shoulder region	Cervical epidural abscess	MRSA	Neck pain, fever → quadriparesis, bladder and bowel dysfunction	Vancomycin → linezolid + teicoplanin	Partial improvement
Turtay, 2014[20]	Turkey	M	51	Chronic back pain	Wet	Lumbar area	Lumbar abscess	N/A	Pain	Drainage and antibiotics (ampicillin-sulbactam + ciprofloxacin)	Complete resolution
Lee, 2014 [24]	Korea	F	59	Abdomen	Dry	Constipation	Superficial skin abscess	*Mycobacterium massiliense*	Skin lesion, discomfort	Wide excision and antibiotics → *en bloc* resection + clarithromycin	Complete resolution
Lee, 2012 [22]	Korea	F	47	Persistent headache	Wet	Posterior nuchal region	Cervical epidural abscess	N/A	Pain, swelling, fever	Vancomycin + co-amoxiclav	Complete resolution
Jung, 2011 [23]	Korea	F	56	left elbow pain	Wet	Left forearm	HSV infection	HSV	Pain, skin lesions	N/A	Complete resolution

*Abbreviations: F* Female, *M* Male, *N/A* Not available, *MRSA* Methicillin-resistant Staphylococcus aureus, *MSSA* Methicillin-sensitive *Staphylococcus aureus*

Seven patients had undergone wet cupping before developing an infection, with three exceptions. Lee et al. [21] reported a case of cutaneous nontuberculous mycobacteria (NTM) infection after dry cupping. Together with aggravation of the condition one year after cupping therapy, a causal link between infection and cupping therapy was debatable in that case. Jung et al. [23] reported a case of herpes simplex virus (HSV) infection after cupping and acupuncture, without describing further details. Wang et al. [16] reported a case of L4-L5 discitis and epidural abscess associated with dry cupping.

In nine cases mentioning the timing of symptom onset, discomfort appeared after cupping therapy within one week. Common complaints were local pain (8/10), swelling (5/10), and obvious skin lesions (3/8) near the sites of cupping. Except for fever, the vital signs on admission were unremarkable in all cases.

Various causative organisms were identified including *Staphylococcus aureus*, *Pseudomonas*, *Mycobacterium massiliense*, *Stenotrophomonas maltophilia*, and HSV. Surgery was performed in four cases, and the remaining cases were treated conservatively. Among nine cases with bacterial infection, the therapeutic regimens were altered according to the results of microbiology and drug sensitivity testing in five cases, with empirical therapy given throughout treatment in others.

Overall, outcomes were favorable in these patients, with full recoveries in all cases apart from one partial recovery and one death; both of these cases were offered surgery but the patients refused. The cause of death in the patient who developed *Pseudomonas-*positive necrotizing fasciitis following wet cupping therapy was largely attributable to the delay in hospital presentation and patient preference for medical management: he presented to the hospital with severe pain and swelling of the lower extremities bilaterally and a high fever two weeks after symptom onset and refused any form of surgical intervention [14].

## Discussion and conclusions

Cupping therapy is generally considered a safe procedure, especially dry cupping, which is noninvasive. Although wet cupping is invasive, it is as safe, if not safer, than simple acupuncture, considering the shorter needle retention time and negative pressure environment in the cups. The risk of serious AEs associated with acupuncture is estimated to be 0.05 per 10,000 treatments [24], lower than many common medical interventions [25].

Al Bedah et al. [10] evaluated the safety of cupping therapy based on six randomized controlled trials (RCTs), 16 case reports, and three case series of cupping therapy from 2000 to 2016. The most frequent AEs were scar formation and burning. Only four cases of cervical and lumber abscess formation and skin infections with HSV and *Mycobacterium* were reported in the review (all included in our review above). Interestingly, all four cases were presented in case reports, but the RCTs reported no similar AEs. Cao et al. [7] reviewed 135 cupping therapy-related RCTs and similarly did not find reports of serious AEs. There are two possible explanations for this. First, in RCTs, cupping therapy is usually performed by trained medical practitioners and follows safety guidelines, while in case reports practitioners of cupping therapy are diverse, even including the patients themselves and unqualified individuals. Infection associated with cupping therapy in these cases is, therefore, probably due to not following best practice. Second, a cross-sectional survey of Korean medical practitioners reported that 20% had patients suffer infections related to wet cupping [26]. Except for the possibility of recall or observer bias, these contradictory results imply that the low reported incidence of AEs might reflect inherent bias in the reporting of RCTs. Also, not all the AEs related to cupping therapy are recorded or published, since there are no rigorous AE registration systems for cupping [11]. Furthermore, we have not found any case reports of hepatitis secondary to cupping therapy, even though several studies have reported that acupuncture and cupping are risk factors for HCV infection in Egypt [27–29] and HDV infection in Iran [30], presumably through transmission from infected needles.

Medical devices [25], the patient’s own skin, and the practitioner’s hands [24] have all been implicated as sources of infection. Koh et al. [31] reported an outbreak of infection related to acupuncture attributable to a contaminated batch of diluted glutaraldehyde solution used for disinfecting medical devices. However, no direct evidence of the source of infection was presented in most cases. In our case, based on an infection developing, we speculate that the wet cupping practitioner had not adhered to high hygiene standards, which is relatively common in TCM practice [32]. The pathogens might have been either directly introduced into the muscle and/or developed as a hematogenous complication, although distinguishing between these two possibilities was difficult because there was both local (iliopsoas abscess) and distant (lung cavitation) involvement.

In any infection related to cupping therapy, it is vitally important to identify the causative organisms and perform drug sensitivity testing. In our case, the patient might have benefited from an earlier change from vancomycin to cefmetazole plus levofloxacin, i.e., broad-spectrum to pathogen-specific antibiotics. Due to the prevalence of MRSA, patients with suspected staphylococcal bacteremia are empirically treated with an anti-MRSA agent (usually vancomycin) until MRSA infection is excluded. However, for patients with MSSA bacteremia, even though whether vancomycin and/or β-lactams should be used as empiric therapy remains under debate, definitive therapy with vancomycin therapy is obviously associated with a higher risk of morbidity and mortality than other β-lactams [33–36], as confirmed by Blumenthal et al. [37] in their decision tree with sensitivity analyses in patients with MSSA bacteremia and reported penicillin allergy. Therefore, deescalating empirical vancomycin as soon as blood culture susceptibilities became available was probably sensible in our case. The majority of reviewed cases chose antibiotic regimens specific to the causative organisms and thus arrested or reversed the disease, again highlighting the need to accurately identify the pathogens and target subsequent therapy.

Two of the reported cases had poor outcomes, largely attributable to refusal of necessary surgical interventions by the affected patients [14, 19]. Both clinicians and patients should all be aware of the potentially serious consequences of infection secondary to cupping therapy and the need for radical management where necessary.

Although Dlaska et al. [38] reported disseminated MSSA infection resulting from a paracervical abscess after acupuncture, the infection was most likely due to unusually long needle retention time. Our case provides rare evidence of disseminated MSSA infection secondary to wet cupping in routine practice. Together with the other ten cases of infective sequelae from cupping, we emphasize that cupping therapy, a relatively safe procedure, can trigger life-threatening infection.

In conclusion, practitioners of cupping therapy, clinicians, and the public should be alert to the risk of infection after cupping therapy. Safety standards and techniques, including hand hygiene, disinfection of the cupping sites, and the use of single-use disposable needles and strictly sterilized medical devices, need to be strictly followed. Patients receiving cupping therapy should be counseled about the risk of infection during the consent procedure and advised to seek urgent medical intervention if they experience persistent pain, swelling, or skin lesions near the cupping sites. Clinicians must promptly recognize this complication, administer pathogen-specific treatment, and provide a strong recommendation to patients to accept the most appropriate treatments.

## Data Availability

All data generated or analysed during this study are included in this published article.

## Abbreviations

TCM: Traditional and complementary medicine
AEs: Adverse events
MSSA: Methicillin-sensitive *Staphylococcus aureus*
CNKI: Chinese National Knowledge Infrastructure
F: Female
M: Male
N/A: Not available
MRSA: Methicillin-resistant *Staphylococcus aureus*
NTM: Nontuberculous mycobacteria
HSV: Herpes simplex virus
RCTs: Randomized controlled trials

## References

[1] Moura C de C, Chaves É de CL, Cardoso ACLR, Nogueira DA, Corrêa HP, Chianca TCM. Cupping therapy and chronic back pain: systematic review and meta-analysis. Rev Lat Am Enfermagem. 2018;26:e3094. 10.1590/1518-8345.2888.3094 PMC6248735 30462793

[2] Mazhar Uddin SM, Haq A, Sheikh H (2016). The use of Hijama (Wet Cupping) in alternative and complementary medicine: efficacious or perilous?. J Acupunct Meridian Stud.

[3] Al Bedah AMN, Khalil MKM, Posadzki P, Sohaibani I, Aboushanab TS, AlQaed M (2016). Evaluation of wet cupping therapy: systematic review of randomized clinical trials. J Altern Complement Med N Y N.

[4] Abdulah DM, Mohammedsadiq HA, Mohammed AH (2021). Effectiveness of wet cupping therapy on relieving pain in patients with chronic migraine: an observational study. J Complement Integr Med.

[5] Ahmadi A, Schwebel DC, Rezaei M (2008). The efficacy of wet-cupping in the treatment of tension and migraine headache. Am J Chin Med.

[6] Cao H, Han M, Li X, Dong S, Shang Y, Wang Q (2010). Clinical research evidence of cupping therapy in China: a systematic literature review. BMC Complement Altern Med.

[7] Cao H, Li X, Liu J (2012). An updated review of the efficacy of cupping therapy. PLoS ONE.

[8] Mehta P, Dhapte V (2015). Cupping therapy: a prudent remedy for a plethora of medical ailments. J Tradit Complement Med.

[9] Al-Bedah AMN, Elsubai IS, Qureshi NA, Aboushanab TS, Ali GIM, El-Olemy AT (2018). The medical perspective of cupping therapy: effects and mechanisms of action. J Tradit Complement Med.

[10] Al-Bedah A, Aboushanab T, Sohaibani I, Ali G, Khalil M, Qureshi N (2016). Safety of cupping therapy in studies conducted in twenty one century: a review of literature. Br J Med Med Res.

[11] Kim T-H, Kim KH, Choi J-Y, Lee MS (2014). Adverse events related to cupping therapy in studies conducted in Korea: a systematic review. Eur J Integr Med.

[12] Ersoy S, Altinoz E, Benli AR, Erdemli ME, Aksungur Z, Gozukara Bag H, et al. Investigation of wet cupping therapy’s effect on oxidative stress based on biochemical parameters. Eur J Integr Med. 2019;30:100946.

[13] Kim J-I, Kim T-H, Lee MS, Kang JW, Kim KH, Choi J-Y (2011). Evaluation of wet-cupping therapy for persistent non-specific low back pain: a randomised, waiting-list controlled, open-label, parallel-group pilot trial. Trials.

[14] Alajmi T, Aljulaihim A, Alzahrani M, Aljuhayyiam S. Necrotizing fasciitis following wet cupping: a case report. Cureus. 2021;13:e14039. 10.7759/cureus.14039 PMC8059418 33898125

[15] El Hasbani G, Jawad A, Uthman I (2020). Septic arthritis of the knee caused by cupping (Hijama). J R Coll Physicians Edinb.

[16] Wang G, Xu N, Yang L, Zheng F, Sai L, Zhou J (2019). Community acquired Stenotrophomonas maltophilia discitis: diagnosis aided by shotgun metagenomic sequencing. Int J Infect Dis.

[17] Xu Z, Sun Y, Li Q, Li J (2019). Xiong Suo Guan Jie Gan Ran 1 Li [A case of sternoclavicular joint infection]. Lin Chuang Gu Ke Za Zhi.

[18] Lu K, Hu H (2017). Zu Bei Ba Guan Zhi Liao Hou Zhi Pi Fu Huai Si Chuang Kou Gan Ran 1 li De Hu Li [Nursing care of skin necrosis and wound infection after cupping on the dorsum of the foot]. Hu Li Yu Kang Fu.

[19] Yao Y, Hong W, Chen H, Guan Q, Yu H, Chang X (2016). Cervical spinal epidural abscess following acupuncture and wet-cupping therapy: A case report. Complement Ther Med.

[20] Turtay MG, Turgut K, Oguzturk H (2014). Unexpected lumbar abscess due to scarification wet cupping: a case report. Complement Ther Med.

[21] Lee SY, Sin JI, Yoo HK, Kim TS, Sung KY (2014). Cutaneous Mycobacterium massiliense infection associated with cupping therapy. Clin Exp Dermatol.

[22] Lee J-H, Cho J-H, Jo D-J (2012). Cervical epidural abscess after cupping and acupuncture. Complement Ther Med.

[23] Jung Y-J, Kim J-H, Lee H-J, Bak H, Hong SP, Jeon SY (2011). A herpes simplex virus infection secondary to acupuncture and cupping. Ann Dermatol.

[24] White A (2004). A cumulative review of the range and incidence of significant adverse events associated with acupuncture. Acupunct Med J Br Med Acupunct Soc.

[25] Guevara-Patiño A, de Mora MS, Farreras A, Rivera-Olivero I, Fermin D, de Waard JH (2010). Soft tissue infection due to Mycobacterium fortuitum following acupuncture: a case report and review of the literature. J Infect Dev Ctries.

[26] Lee B, Song Y, Lim H (2008). Literature investigation regarding cupping therapy and analysis of current professional’s cupping treatment. J Orient Rehab Med.

[27] Heiza M, Elmola K, Salama B (2021). Unsafe practices associated with HCV infection among adults: a case control study. Int J Prev Med.

[28] W Abd El-Wahab E, Mikheal A, Sidkey F, Shatat HZ (2014). Factors associated with hepatitis C infection among chronic HCV Egyptian patients. Iran J Public Health.

[29] El-Ghitany EM, Abdel Wahab MM, Abd El-Wahab EW, Hassouna S, Farghaly AG (2015). A comprehensive hepatitis C virus risk factors meta-analysis (1989–2013): do they differ in Egypt?. Liver Int Off J Int Assoc Study Liver.

[30] Ghadir M-R, Belbasi M, Heidari A, Sarkeshikian SS, Kabiri A, Ghanooni AH (2012). Prevalence of hepatitis D virus infection among hepatitis B virus infected patients in Qom Province. Center of Iran Hepat Mon.

[31] Koh S-J, Song T, Kang YA, Choi JW, Chang KJ, Chu CS (2010). An outbreak of skin and soft tissue infection caused by Mycobacterium abscessus following acupuncture. Clin Microbiol Infect.

[32] Song G (2015). Problem of nosocomial infection in acupuncture and moxibustion treatment and its solutions. West J Tradit Chin Med.

[33] Wong D, Wong T, Romney M, Leung V (2016). Comparative effectiveness of β-lactam versus vancomycin empiric therapy in patients with methicillin-susceptible Staphylococcus aureus (MSSA) bacteremia. Ann Clin Microbiol Antimicrob.

[34] Wong D, Wong T, Romney M, Leung V (2016). Comparison of outcomes in patients with methicillin-susceptible Staphylococcus aureus (MSSA) bacteremia who are treated with β-lactam vs vancomycin empiric therapy: a retrospective cohort study. BMC Infect Dis.

[35] McDanel JS, Perencevich EN, Diekema DJ, Herwaldt LA, Smith TC, Chrischilles EA (2015). Comparative effectiveness of beta-lactams versus vancomycin for treatment of methicillin-susceptible Staphylococcus aureus bloodstream infections among 122 hospitals. Clin Infect Dis Off Publ Infect Dis Soc Am.

[36] McConeghy KW, Bleasdale SC, Rodvold KA (2013). The empirical combination of vancomycin and a β-lactam for staphylococcal bacteremia. Clin Infect Dis.

[37] Blumenthal KG, Parker RA, Shenoy ES, Walensky RP (2015). Improving clinical outcomes in patients with methicillin-sensitive staphylococcus aureus bacteremia and reported penicillin allergy. Clin Infect Dis.

[38] Dlaska CE, Temple S, Schuetz MA (2015). Disseminated methicillin-sensitive staphylococcus aureus infection resulting from a paracervical abscess after acupuncture. Med J Aust.

